# Factors Associated with Therapeutic Alliance Among Outpatient Musculoskeletal Physiotherapy Patients in Saudi Arabia: A Cross-Sectional Study

**DOI:** 10.3390/healthcare14203402

**Published:** 2026-10-12

**Authors:** Neah Albasha, Dalia Binshaye, Daad Alhumaid, Fatimah Almohsen, Rahaf Alshareef, Najd Alkhushayban, Sarah Mohammed Alsubaie, Fatmah Hasani, Samiah Alqabbani

**Affiliations:** 1 Department of Rehabilitation Sciences, College of Health and Rehabilitation Sciences, Princess Nourah Bint Abdulrahman University, Riyadh 11671, Saudi Arabia442005007@pnu.edu.sa (D.B.); 442001888@pnu.edu.sa (S.M.A.); 2 Medical Cities Program, General Directorate of Medical Services, Ministry of Interior, Riyadh 12812, Saudi Arabia

**Keywords:** therapeutic alliance, physiotherapy, self-efficacy, quality of life, biopsychosocial model, rehabilitation

## Abstract

Background: Therapeutic alliance is an important component of physiotherapy rehabilitation and is associated with patient engagement, perceived improvement, and rehabilitation outcomes. However, limited evidence exists regarding factors associated with therapeutic alliance among physiotherapy patients in Saudi Arabia. Objective: To examine therapeutic alliance among adult outpatient musculoskeletal physiotherapy patients in Saudi Arabia and investigate its association with self-efficacy, quality of life, pain-related variables, and treatment-related factors. Methods: A cross-sectional study was conducted among adult outpatient musculoskeletal physiotherapy patients in Saudi Arabia. Data were collected using an electronic questionnaire including demographic and clinical characteristics, the Working Alliance Inventory–Short Form, General Self-Efficacy Scale, WHOQOL-BREF, and pain-related measures. Data were analyzed using descriptive statistics, Spearman’s rho correlation, Mann–Whitney U test, Kruskal–Wallis test, and hierarchical multiple regression. Results: A total of 174 participants were included. Participants reported generally high therapeutic alliance scores, with a median WAI total score of 57.0 (IQR = 8.0). Therapeutic alliance was positively associated with self-efficacy, quality of life domains, and perceived improvement (all *p* < 0.05). Participants treated by the same physiotherapist across sessions and those receiving longer treatment sessions reported significantly higher therapeutic alliance scores (both *p* < 0.05). The final model explained 40.6% of the variance in therapeutic alliance scores. Higher self-efficacy, greater perceived improvement, longer session duration, higher environmental quality of life, and treatment by the same physiotherapist across sessions were positively associated with higher therapeutic alliance scores. Conclusion: Therapeutic alliance among outpatient physiotherapy patients was associated with both patient-related and treatment-related factors, supporting the importance of a biopsychosocial and patient-centered approach in physiotherapy rehabilitation. Contribution of the paper: Limited research has examined the factors associated with therapeutic alliance in physiotherapy within an integrated biopsychosocial framework. This study identified psychosocial, symptom-related, environmental, and treatment-related factors associated with therapeutic alliance among outpatient musculoskeletal physiotherapy patients. Self-efficacy, perceived improvement, environmental quality of life, session duration, and therapist continuity emerged as important factors associated with therapeutic alliance, highlighting potential targets for patient-centered physiotherapy care.

## 1. Introduction

Effective physiotherapy relies not only on the selection of appropriate interventions, but also on patients’ engagement, communication, and active participation throughout rehabilitation [1]. Consequently, growing attention has been directed toward the role of therapeutic alliance (TA) within physiotherapy practice. TA refers to the collaborative relationship between the patient and physiotherapist, including agreement on treatment goals, therapeutic tasks, and the development of trust and rapport [2,3]. Despite the growing evidence linking TA to rehabilitation outcomes, considerably less attention has been directed toward understanding the factors associated with the development of TA within physiotherapy settings.

The biopsychosocial framework originally proposed by Engel recognizes that health and illness are not solely determined by biological factors but are also shaped by psychological and social influences [4]. This framework is particularly relevant to physiotherapy, where the TA may develop through the interplay between patients’ psychological resources, social relationships, physical condition, broader environmental context, and experiences of treatment. Accordingly, TA may be associated with multiple psychosocial, clinical, environmental, and treatment-related factors. Patient-level psychosocial factors, including patients’ self-efficacy (SE) and psychological and social quality of life (QoL), reflect patients’ psychological resources and interpersonal context, which may be related to their confidence, motivation, and ability to actively engage in physiotherapy and communicate effectively with their physiotherapist [5,6]. Similarly, clinical status and perceived treatment factors, represented by pain intensity, physical QoL, and perceived improvement, capture patients’ physical functioning, symptom burden, and perceptions of progress during rehabilitation. These factors may be related to how patients perceive the effectiveness and relevance of treatment and, consequently, to their engagement with the therapeutic process. Treatment-related factors, including the number and duration of sessions and continuity with the same physiotherapist, may provide opportunities for repeated interaction, trust, reassurance, and collaborative engagement throughout treatment [7,8]. Environmental aspects of patients’ quality of life may also be associated with rehabilitation experiences through factors related to healthcare access, transportation, safety, financial resources, and the home environment [9]. Accordingly, these factors can be conceptually organized into patient-level psychosocial factors, clinical status and perceived improvement, and broader environmental and treatment-related factors. Within this framework, environmental and treatment-related factors represent the wider context in which rehabilitation occurs, including the conditions that may influence patients’ access to, experience of, and participation in physiotherapy beyond their psychological, social, and physical health status.

Although therapeutic alliance is increasingly recognized as an important component of physiotherapy rehabilitation, limited research has comprehensively examined the multiple factors associated with TA within an integrated biopsychosocial framework. Existing physiotherapy literature has often focused on isolated psychological or treatment-related variables, with less attention directed toward how psychosocial, physical, environmental, and treatment-related factors are collectively associated with therapeutic alliance. This gap is particularly important within physiotherapy rehabilitation, where long-term patient engagement, communication, adherence, and collaborative participation are central components of care.

Therefore, guided by the biopsychosocial framework, this study aimed to investigate therapeutic alliance among physiotherapy patients and to examine the associations of theoretically organized groups of factors: patient-level psychosocial factors, clinical status and perceived improvement, and broader environmental and treatment-related factors.

## 2. Methods

### 2.1. Study Design, Participants, and Sampling

This cross-sectional study used a convenience sampling strategy to examine therapeutic alliance and its associated biopsychosocial and treatment-related factors among adult physiotherapy patients. Participants were recruited primarily through three outpatient physiotherapy centers, with additional electronic distribution through physiotherapy networks reaching patients attending other public and private outpatient facilities in Saudi Arabia through electronic distribution of the survey between March and May 2026. Responses were screened for identical demographic and response patterns, and no apparent duplicates were identified. Eligible participants were adults aged 18 years or older who were currently receiving outpatient physiotherapy for a musculoskeletal condition and were able to communicate in Arabic. Participants were excluded if they were not currently receiving outpatient physiotherapy, were receiving physiotherapy for non-musculoskeletal conditions, or self-reported cognitive impairment or psychiatric conditions that could interfere with survey completion. Eligibility was verified through screening questions at the beginning of the survey.

A priori sample size calculation was conducted using G*Power version 3.1 for fixed-model multiple regression, assuming a medium effect size (f^2^ = 0.15), α = 0.05, statistical power of 95%, and 10 independent factors, the minimum required sample size was 172 participants.

### 2.2. Measure and Study Variables

Data were collected using an electronic survey developed in Microsoft Forms (Microsoft Corporation, Redmond, WA, USA). The survey began with an informed consent page, and participants were required to provide electronic consent before proceeding. Eligible participants completed sections covering demographic characteristics, biopsychosocial variables, treatment-related factors, therapeutic alliance, self-efficacy, and quality of life. Prior to data collection, the survey was reviewed and pilot tested by the research team to ensure clarity, comprehensibility, and logical flow. Demographic variables included age, gender, marital status, education, and income. Physical and treatment-related variables included diagnosis, comorbidities, previous physiotherapy experience, number of completed sessions, therapist continuity, facility type, session frequency and duration, and payment type. Therapist continuity was assessed using a single yes/no question asking whether the participant had been treated by the same physiotherapist in all sessions. A “yes” response indicated continuity across all sessions received up to the time of survey completion.

Pain intensity was assessed using the Arabic Numeric Pain Rating Scale, an 11-point scale ranging from 0 (“no pain”) to 10 (“worst imaginable pain”), which has demonstrated acceptable validity and reliability in Arabic-speaking patients with musculoskeletal conditions.

Perceived improvement was assessed using a single study-specific item asking participants to rate their overall improvement since starting physiotherapy. Response options were worse, no change, some improvement, substantial improvement, and full recovery, with higher categories indicating greater perceived improvement.

Therapeutic alliance was assessed using the Working Alliance Inventory–Short Form (WAI-S), a 12-item measure assessing agreement on goal, therapeutic tasks, and therapeutic bond. Items are rated on a 5-point Likert scale from 1 (“seldom”) to 5 (“always”), with higher scores indicating stronger therapeutic alliance. The total score ranges from 12 to 60, with higher scores indicating stronger therapeutic alliance [3]. Each subdomain—Goal, Task, and Bond—contains four items. Subdomain scores were calculated as the mean of the four corresponding items, resulting in a possible score ranging from 1 to 5. The Arabic version was obtained with permission from the Society for Psychotherapy Research, including permission for adaptation to the physiotherapy context. Face validity was assessed through interviews with Arabic-speaking physiotherapy participants to evaluate clarity, comprehensibility, wording, and cultural appropriateness. Minor linguistic and contextual modifications were made based on patient feedback to improve the clarity and applicability of the statements to physiotherapy practice while preserving the original conceptual meaning of the instrument. For example, the statement referring to working together to achieve treatment goals was revised to emphasize collaborative goal settings in physiotherapy. Additionally, selected statements addressing patients’ perceptions of change and treatment activities were reworded to enhance their clarity and relevance to physiotherapy sessions.

Content validity was evaluated by a seven-member expert panel, including four licensed physiotherapists, two academic researchers in rehabilitation and health sciences, and one language expert. The scale-level content validity index based on the average method (S-CVI/Ave) was 0.88, with subscale values of 0.93 for Goal, 0.82 for Task, and 0.88 for Bond.

The internal consistency was initially examined in an independent pilot sample of 25 participants, yielding a Cronbach’s alpha of 0.77. Internal consistency was assessed in the main study sample (N = 174), demonstrating Cronbach’s alpha coefficient of 0.91 for the total scale, 0.84 for Goal, 0.85 for Task, and 0.70 for Bond.

Self-efficacy was assessed using the Arabic version of the General Self-Efficacy Short Scale-3 (GSE-3). The GSE-3 was developed as an English-language adaptation by Doll et al. [10], and its Arabic psychometric properties were subsequently evaluated by Fekih-Romdhane et al. [11]. The scale score was calculated as the mean of the three items, resulting in a possible score ranging from 1 to 5, with higher scores indicating greater general self-efficacy. Permission to use the Arabic version was obtained before data collection. In the present sample, the scale demonstrated excellent internal consistency (Cronbach’s α = 0.94).

Quality of life (QoL) was assessed using the Arabic version of the WHOQOL-BREF, a 26-item self-report questionnaire covering four domains: physical health, psychological health, social relationships, and environment. Items are rated on a 5-point Likert scale, and domain scores were calculated by averaging the corresponding items, and the composite QoL score was calculated as the average of the four domain scores, with higher scores indicating better quality of life [12].

### 2.3. Ethics

Ethical approval was obtained from Princess Nourah bint Abdulrahman University Institutional Review Board (IRB) prior to data collection (IRB Log Number: 26-0100). Electronic informed consent was obtained from all participants before participation in the study. Participation was voluntary, and all responses were collected anonymously and kept confidential throughout the research process.

### 2.4. Statistical Analysis

Data were analyzed using IBM SPSS Statistics version 31 (IBM Corp., Armonk, NY, USA). Descriptive statistics for categorical variables were reported as frequencies and percentages, while continuous or ordinal variables were reported as median and interquartile ranges. Normality was assessed prior to inferential analyses. Associations between therapeutic alliance and continuous variables were examined using Spearman’s rho correlation. Differences in WAI-S scores across categorical variables were assessed using the Mann–Whitney U test for two-group comparisons and the Kruskal–Wallis test for variables with more than two groups, followed by Bonferroni-adjusted post hoc pairwise comparisons where appropriate. Internal consistency was assessed using Cronbach’s alpha. Hierarchical multiple regression analysis was conducted to identify factors associated with therapeutic alliance. Variables included in the regression were selected a priori based on the study’s conceptual framework, hypotheses, and clinical relevance. Other demographic and service-related characteristics presented in Table 1 were reported descriptively and were not screened for inclusion based on bivariate statistical significance. Before conducting the hierarchical multiple regression analysis, session duration was dummy coded, with sessions lasting less than 30 min used as the reference category. Perceived improvement was coded from 0 (worse) to 4 (full recovery) and retained as an ordinal variable reflecting progressively higher levels of perceived improvement. Its coefficient represents the average change in the WAI total score associated with a one-category increase in perceived improvement. The ordinal trend specification was retained because the categories represented progressively higher levels of perceived improvement, whereas separate indicator coding would require additional parameters estimated from categories with few or no observations. Residual normality, linearity, homoscedasticity, independence of errors, multicollinearity, and influential observations were assessed using residual plots and relevant diagnostic statistics. The residuals were approximately normally distributed, and the regression assumptions were adequately met. Variables were entered sequentially according to their conceptual relevance. Model 1 included psychosocial factors; Model 2 additionally incorporated pain intensity, perceived improvement, and physical quality-of-life factors, while Model 3 further included environmental and treatment-related factors. Statistical significance was determined at a *p*-value of <0.05.

## 3. Results

A total of 258 responses were initially received. Eighty-four respondents were excluded because they were not currently receiving physiotherapy sessions, leaving 174 participants receiving outpatient physiotherapy for musculoskeletal conditions in the final analysis. Participant demographic, symptom-related, and treatment-related characteristics are presented in Table 1. Most participants were female (66.1%), and the most represented age group was 45–64 years (36.2%). Lower limb conditions were the most frequently reported diagnosis (31.0%). Presence of comorbidity was reported by 36.2% of participants, 85.6% had previous physiotherapy experience, and 54.0% were receiving physiotherapy for their current diagnosis for the first time. Most participants reported receiving treatment from the same physiotherapist across sessions (89.1%), and sessions lasting more than 30 min were the most reported duration category (45.4%).

### 3.1. Therapeutic Alliance, Self-Efficacy, Quality of Life, and Pain-Related Variables

Descriptive analysis of the main study variables showed generally high therapeutic alliance scores among participants, with a median WAI total score of 57.00 (IQR = 8.00). Across the WAI subdomains, the Goal domain demonstrated the highest median score (median = 5.00, IQR = 0.50), followed by the Bond domain (median = 4.75, IQR = 0.75) and the Task domain (median = 4.75, IQR = 1.00).

Self-efficacy scores were generally high among participants, with a median score of 4.00 (IQR = 2.00). Quality-of-life scores were also relatively favorable, with a median composite QoL score of 3.71 (IQR = 0.67). Across the QoL domains, social domain demonstrated the highest median score (median = 4.00, IQR = 0.67), followed by the psychological domain (median = 3.80, IQR = 1.00), environmental domain (median = 3.75, IQR = 0.75), and physical domain (median = 3.43, IQR = 1.00).

The median pain intensity score was 4.00 (IQR = 6.00), indicating moderate pain levels overall. Regarding perceived improvement, 4.6% of participants reported no change, 33.9% reported some improvement, 55.7% reported substantial improvement, and 5.7% reported full recovery following physiotherapy treatment.

### 3.2. Associations Between TA and Patient-/Treatment-Related Factors

Therapeutic alliance demonstrated significant associations with several patient-related and treatment-related factors (Table 2). Higher WAI total scores were significantly associated with greater self-efficacy (ρ = 0.29, *p* < 0.001), environmental quality of life (ρ = 0.30, *p* < 0.001), psychological quality of life (ρ = 0.21, *p* = 0.006), social quality of life (ρ = 0.19, *p* = 0.011), physical quality of life (ρ = 0.17, *p* = 0.022), and perceived improvement (ρ = 0.44, *p* < 0.001). Pain intensity demonstrated a significant negative association with therapeutic alliance (ρ = −0.26, *p* = 0.001). No significant association was observed between therapeutic alliance and number of physiotherapy sessions (*p* = 0.245).

Regarding treatment-related variables, participants treated by the same physiotherapist across sessions reported significantly higher WAI scores than those treated by different physiotherapists (*p* = 0.014). Therapeutic alliance also differed significantly according to session duration (H = 11.906, *p* = 0.003), Bonferroni-adjusted post hoc comparisons showed significantly higher WAI scores among participants receiving 30 min sessions compared with those receiving sessions lasting less than 30 min (adjusted *p* = 0.011), and among those receiving sessions lasting more than 30 min compared with those receiving sessions lasting less than 30 min (adjusted *p* = 0.002). No significant difference was observed between the 30 min and more-than-30 min groups (adjusted *p* = 1.000) (Table 3).

### 3.3. Hierarchical Regression Analysis

Hierarchical multiple regression analysis was conducted to identify factors independently associated with therapeutic alliance. The Durbin–Watson statistic was 2.237, VIF values ranged from 1.067 to 2.785, tolerance values ranged from 0.359 to 0.937, the maximum Cook’s distance was 0.164, and standardized residuals ranged from −2.438 to 2.197. Overall, these diagnostic values were considered acceptable. The final regression model explained 40.6% of the variance in therapeutic alliance scores (R^2^ = 0.406, adjusted R^2^ = 0.366). Detailed results are presented in Table 4.

The addition of psychosocial variables in Model 1 explained 13.0% of the variance in therapeutic alliance scores, with self-efficacy (β = 0.26, *p* < 0.001) and the social quality-of-life domain (β = 0.18, *p* = 0.037) emerging as significant positive factors associated with therapeutic alliance. Incorporating pain intensity, perceived improvement, and physical quality-of-life variables in Model 2 explained an additional 18.9% of the variance (ΔR^2^ = 0.189, *p* < 0.001), increasing the total explained variance to 31.9%. In this model, self-efficacy (β = 0.19, *p* = 0.007) and perceived improvement (β = 0.43, *p* < 0.001) remained significant independent factors associated with therapeutic alliance, whereas pain intensity and the physical, psychological, and social quality-of-life domains were not independently associated with WAI total scores.

The addition of environmental and treatment-related variables in Model 3 explained a further 8.8% of the variance (ΔR^2^ = 0.088, *p* < 0.001). In the final model, self-efficacy (β = 0.14, *p* = 0.035), perceived improvement (β = 0.38, *p* < 0.001), a session duration of 30 min (β = 0.22, *p* = 0.033), a session duration of more than 30 min (β = 0.32, *p* = 0.002), environmental quality of life (β = 0.27, *p* = 0.007), and treatment continuity with the same physiotherapist (β = 0.16, *p* = 0.014) were independently associated with therapeutic alliance. Current pain intensity, number of physiotherapy sessions, and the physical, psychological, and social QoL domains were not significant factors in the final model.

## 4. Discussion

This study examined biopsychosocial and treatment-related factors associated with therapeutic alliance among adult outpatient physiotherapy patients in Saudi Arabia. Overall, participants reported high therapeutic alliance scores, suggesting generally positive patient–physiotherapist relationships. Therapeutic alliance was associated with several patient-related and treatment-related factors, including self-efficacy, quality of life, pain intensity, perceived improvement, therapist continuity, and session duration. Collectively, these findings suggest that therapeutic alliance is associated with a combination of psychological, physical, environmental, and treatment-related factors.

Physiotherapy as a profession is inherently interactive and patient-centered, involving repeated contact between patients and physiotherapists throughout the rehabilitation process [13]. This ongoing interaction commonly includes communication, patient education, shared goal setting, exercise progression, and continuous monitoring of recovery [8]. The generally high therapeutic alliance scores observed in the present study may therefore reflect the relational and collaborative nature of physiotherapy rehabilitation itself. Similar findings have been reported in previous musculoskeletal physiotherapy research, where patients commonly describe positive therapeutic relationships characterized by communication, trust, and collaborative engagement throughout rehabilitation [14,15]. Moreover, Alodaibi et al. [16] similarly reported high alliance scores among patients receiving physiotherapy for musculoskeletal conditions.

Beyond the overall high alliance scores, the findings further demonstrated that therapeutic alliance was associated with several interacting patient-related and treatment-related factors. Psychosocial factors, particularly self-efficacy and multiple domains of quality of life, demonstrated positive associations with therapeutic alliance. Patients with greater confidence in managing their condition and better perceived well-being may engage more effectively in communication, shared decision-making, and active participation throughout rehabilitation. These findings align with previous physiotherapy literature linking therapeutic alliance with self-belief, patient engagement, and overall well-being during rehabilitation [17,18].

Notably, self-efficacy remained consistently associated with therapeutic alliance even after accounting for symptom-related and treatment-related variables. This finding further supports the psychological dimension of the biopsychosocial framework and suggests that patients with greater confidence in managing health-related challenges may be more likely to develop stronger therapeutic relationships with their physiotherapists [19]. From a rehabilitation perspective, patients with higher self-efficacy may communicate more openly, participate more actively in treatment planning, and engage more consistently in rehabilitation activities and self-management behaviors. Similar observations have been reported in physiotherapy literature, where patient engagement, collaborative communication, and self-management approaches were associated with stronger therapeutic relationships and improved rehabilitation experiences [20,21].

In addition to psychosocial influences, symptom-related experiences also were associated with therapeutic alliances. Greater pain intensity was negatively associated with therapeutic alliance, suggesting that persistent or severe symptoms may be related to differences in patient engagement and perceptions of the therapeutic relationship. Within the biopsychosocial framework, physical symptoms are not experienced in isolation but interact with patients’ emotional responses, recovery expectations, and perceptions of rehabilitation progress [22]. In contrast, perceived improvement showed one of the strongest positive associations with therapeutic alliance. However, given the cross-sectional design, the direction of this association cannot be determined. Perceived improvement may strengthen patients’ confidence and trust in the rehabilitation process, while a stronger therapeutic alliance may also be associated with favorable perceptions of recovery and treatment engagement. This interpretation is consistent with the evidence showing that perceived recovery is related to psychological and rehabilitation-related factors [23]. Similarly, Holmes et al. [24] reported an association between therapeutic alliance and patient-reported outcomes in musculoskeletal conditions, further supporting the possibility of a bidirectional relationship.

Beyond patient-related experiences, treatment-related and contextual factors were also associated with therapeutic alliance. Longer physiotherapy session duration and continuity with the same physiotherapist were both positively associated with therapeutic alliance, highlighting the importance of time, communication, and relational continuity in rehabilitation. Longer sessions may provide greater opportunities for collaborative goal setting, patient education, and trust-building, while continuity with the same physiotherapist may strengthen therapeutic bonding and relational consistency over time [25,26]. These findings are particularly relevant within physiotherapy rehabilitation, where treatment commonly involves repeated interactions and ongoing progression over multiple sessions. Environmental quality of life also remained positively associated with therapeutic alliance, suggesting that broader contextual factors such as healthcare accessibility, financial resources, social support, and living conditions may be related to patients’ ability to engage effectively in rehabilitation.

Taken together, these findings support the biopsychosocial model of rehabilitation by demonstrating that therapeutic alliance in physiotherapy cannot be explained by a single factor alone, but rather reflects the interaction of psychological, physical, environmental, and treatment-related dimensions within the rehabilitation process. The present findings also highlight the importance of considering both patient-level and treatment-related aspects of physiotherapy care when aiming to optimize therapeutic relationships and rehabilitation engagement.

This study has several strengths. It is among the first studies in Saudi Arabia to examine these multidimensional associations within a physiotherapy rehabilitation context using culturally adapted Arabic outcome measures. The study also included multiple patient-related and treatment-related factors and utilized culturally adapted Arabic outcome measures relevant to rehabilitation practice. Several limitations should also be acknowledged. The cross-sectional design limits the ability to determine causal relationships between therapeutic alliance and the associated factors identified in this study. In particular, the relationship between perceived improvement and therapeutic alliance may be bidirectional. Similarly, the associations with session duration and therapist continuity may reflect unmeasured clinical complexity, service organization, or other contextual factors. More broadly, residual confounding by demographic, clinical, or service-related characteristics not included in the regression model cannot be excluded. Data were collected using self-reported electronic questionnaires, which may introduce recall and response bias. Perceived improvement was assessed using a single self-reported item rather than a validated multi-item measure, which may limit measurement precision. Its ordinal coding also assumes that moving from one improvement category to the next represents a similar degree of change, which may not fully reflect the differences between categories. In addition, the use of convenience sampling may limit the generalizability of the findings to all physiotherapy populations and clinical settings. The study also focused primarily on outpatient musculoskeletal physiotherapy patients, which may limit applicability to other physiotherapy specialties and rehabilitation contexts. Although the adapted Arabic WAI-S demonstrated acceptable content validity and internal consistency, further psychometric evaluation is needed to establish its measurement properties in physiotherapy populations. Finally, the sample size was relatively limited in relation to the number of parameters included in the final regression model; therefore, the possibility of model overfitting and imprecise estimates cannot be excluded. Future research should utilize longitudinal and prospective study designs to further examine the causal relationships between therapeutic alliance and rehabilitation outcomes. Additional studies involving different physiotherapy specialties and healthcare settings are also recommended to better understand how therapeutic alliance develops across diverse rehabilitation populations and contexts.

## 5. Conclusions

Therapeutic alliance among outpatient physiotherapy patients in Saudi Arabia was generally strong and was associated with multiple psychosocial, symptom-related, environmental, and treatment-related factors. Self-efficacy, perceived improvement, environmental quality of life, session duration, and continuity with the same physiotherapist emerged as important factors associated with therapeutic alliance. These findings highlight the importance of adopting a biopsychosocial and patient-centered approach within physiotherapy rehabilitation that extends beyond physical interventions alone. Strategies that support communication, continuity of care, patient confidence, and active rehabilitation engagement may be relevant considerations when seeking to support therapeutic alliance in physiotherapy practices. Future longitudinal studies are needed to further examine the causal and longitudinal relationships between therapeutic alliance and rehabilitation outcomes.

## Figures and Tables

**Table 1:** Demographic Characteristics and Treatment-Related Variables of the Participants (N = 174).

Variable	Category	Frequency (Percent)
Gender	Male	59 (33.9)
	Female	115 (66.1)
Age	18–29 years	49 (28.2)
	30–44 years	47 (27.0)
	45–64 years	63 (36.2)
	65 years and above	15 (8.6)
Marital status	Married	96 (55.2)
	Single	60 (34.5)
	Divorced	11 (6.3)
	Widow	7 (4.0)
Education level	Pre-university education	54 (31.2)
	University education	97 (56.1)
	Higher education	22 (12.7)
PT Diagnosis/condition	Spine	32 (18.4)
	Upper limb	35 (20.1)
	Lower limb	54 (31.0)
	Sport	21 (12.1)
	Post-op	18 (10.3)
	Others	14 (8.0)
Presence of comorbidity	Yes	63 (36.2)
	No	111 (63.8)
Payment type	Out-of-pocket payment	43 (24.7)
	Insurance	52 (29.9)
	Government	79 (45.4)
Type of PT setting	Government hospital	82 (47.1)
	Private hospital	85 (48.9)
	Rehabilitation center	7 (4.0)
Previous physiotherapy experience	Yes	149 (85.6)
	No	25 (14.4)
First physiotherapy for current diagnosis	Yes	94 (54.0)
	No	80 (46.0)
Same therapist across sessions	Different therapist	19 (10.9)
	Same therapist	155 (89.1)
Duration of PT session	Less than 30 min	21 (12.1)
	30 min	74 (42.5)
	More than 30 min	79 (45.4)
Session frequency	Once weekly	73 (42.0)
	Twice weekly	62 (35.6)
	Three times weekly	19 (10.9)
	Twice monthly	6 (3.4)
	Once monthly	11 (6.3)
	Others	3 (1.7)
Pain category	Without pain	55 (31.6)
	Mild pain	13 (7.5)
	Moderate pain	65 (37.4)
	Severe pain	41 (23.6)
Perceived Improvement	No change	8 (4.6)
	Some improvement	59 (33.9)
	Substantial improvement	97 (55.7)
	Full recovery	10 (5.7)

“Other” responses were retained for descriptive reporting only; no additional specification was collected, and these categories were not included in the inferential or regression analyses.

**Table 2:** Correlations Between WAI Total Score and Continuous and Ordinal Study Variables (N = 174).

Variable	Spearman’s Rho (ρ)	*p*-Value
Self-efficacy	0.29	<0.001
Psychological QoL	0.21	0.006
Social QoL	0.19	0.011
Physical QoL	0.17	0.022
Environmental QoL	0.30	<0.001
Average pain intensity	−0.26	0.001
Perceived Improvement	0.44	<0.001
Number of sessions	0.09	0.245

WAI = Working Alliance Inventory; *p* = statistical significance.

**Table 3:** Comparison of WAI Total Score Across Treatment-Related Variables (N = 174).

Treatment-Related Variable	Category	Median (IQR)	Mean Rank	Test Statistic	Effect Size	*p*-Value
Same Therapist Across Sessions	Same therapist	57 (7.00)	90.75	U = 968.5	r = 0.19	0.014
	Different therapist	52 (10.00)	60.97			
Session Duration	Less than 30 min	50 (8.50)	53.24	H = 11.906	η^2^ = 0.06	0.003
	30 min	57 (8.25)	87.06			
	More than 30 min	58 (6.00)	95.13			

WAI = Working Alliance Inventory; IQR = Interquartile Range; U = Mann–Whitney U test statistic; r = effect size for the Mann–Whitney U test; H = Kruskal–Wallis test statistic; η^2^ = eta squared effect size for Kruskal–Wallis test.

**Table 4:** Hierarchical Multiple Regression Analysis of factors associated with Therapeutic Alliance (N = 174).

Model statistics	R^2^	Adjusted R^2^	F change	ΔR^2^	*p*
	0.130	0.115	8.470	0.130	<0.001
**Model 1:** Psychological and Social Factors					
**Independent Factors**	**B (95% CI)**	**SE**	**β**	**t**	***p***
Self-efficacy	0.50 (0.23, 0.78)	0.14	0.26	3.63	<0.001
Psychological QoL	0.51 (−1.04, 2.06)	0.79	0.06	0.65	0.52
Social QoL	1.41 (0.08, 2.74)	0.67	0.18	2.10	0.037
**Model 2:** Pain Intensity, Perceived Improvement, and Physical Quality of Life Factors					
**Model statistics**	**R^2^**	**Adjusted R^2^**	**F change**	**ΔR^2^**	***p***
	0.319	0.294	15.412	0.189	<0.001
**Independent Factors**	**B (95% CI)**	**SE**	**β**	**t**	***p***
Self-efficacy	0.36 (0.10, 0.62)	0.13	0.19	2.72	0.007
Psychological QoL	0.54 (−1.03, 2.11)	0.80	0.06	0.68	0.500
Social QoL	0.21 (−1.10, 1.52)	0.66	0.03	0.32	0.752
Current pain intensity	−0.18 (−0.46, 0.11)	0.15	−0.09	−1.21	0.227
Perceived Improvement	3.86 (2.57, 5.16)	0.65	0.43	5.90	<0.001
Physical QoL	0.13 (−1.47, 1.73)	0.81	0.02	0.16	0.874
**Model 3:** Environmental and Treatment-related Factors					
**Model statistics**	**R^2^**	**Adjusted R^2^**	**F change**	**ΔR^2^**	***p***
	0.406	0.366	4.777	0.088	<0.001
**Independent Factors**	**B (95% CI)**	**SE**	**β**	**t**	***p***
Self-efficacy	0.27 (0.02, 0.52)	0.13	0.14	2.12	0.035
Psychological QoL	−0.39 (−2.00, 1.23)	0.82	−0.04	−0.47	0.637
Social QoL	−0.10 (−1.48, 1.28)	0.70	−0.01	−0.14	0.886
Current pain intensity	−0.19 (−0.46, 0.09)	0.14	−0.09	−1.35	0.178
Perceived Improvement	3.42 (2.13, 4.70)	0.65	0.38	5.25	<0.001
Physical QoL	−0.48 (−2.04, 1.08)	0.79	−0.06	−0.60	0.546
Session duration: 30 min	2.62 (0.22, 5.02)	1.22	0.22	2.15	0.033
Session duration: >30 min	3.89 (1.48, 6.29)	1.22	0.32	3.18	0.002
Number of sessions	−0.28 (−0.76, 0.20)	0.24	−0.08	−1.17	0.244
Environmental QoL	2.42 (0.67, 4.16)	0.88	0.27	2.74	0.007
Same therapist across sessions	3.003 (0.62, 5.38)	1.21	0.16	2.49	0.014

B = unstandardized regression coefficient; SE = standard error; β = standardized beta coefficient; ΔR^2^ = change in explained variance. *p* = significant level. For session duration, sessions lasting < 30 min were used as the reference category.

## Data Availability

The datasets generated and/or analyzed during the current study are available from the corresponding authors on reasonable request due to privacy considerations.
